# Static and Dynamic Mechanical Responses and Synergistic Mechanisms of Concrete Modified with Nano-CaCO_3_ and PP Fibers

**DOI:** 10.3390/ma19173606

**Published:** 2026-08-25

**Authors:** Shengquan Zhou, Zhiqiang Lin, Jiaming Li, Zhaibang Ke, Yongfei Zhang

**Affiliations:** 1 School of Civil Engineering and Architecture, Anhui University of Science and Technology, Huainan 232001, China; lqpzsq@163.com (S.Z.); 15956729015@163.com (J.L.); 2 Anhui Construction Engineering Group Sanjian Co., Ltd., Hefei 231131, China; kezb6789@126.com; 3 Department of Civil Engineering, Faculty of Civil Engineering and Mechanics, Kunming University of Science and Technology, Kunming 650500, China

**Keywords:** impact-resistant concrete, split Hopkinson pressure bar, nano-calcium carbonate, polypropylene fiber, synergistic mechanism

## Abstract

To investigate the synergistic effects and underlying mechanisms of nano-CaCO_3_ (NCC) and polypropylene fibers (PPF) on the static and dynamic mechanical properties of concrete, this study systematically examines hybrid-modified concrete with varying additive contents. Compressive strength, splitting tensile strength, and split Hopkinson pressure bar (SHPB) tests were conducted to evaluate the mechanical responses under quasi-static and high-strain-rate impact loadings. Additionally, scanning electron microscopy (SEM), X-ray diffraction (XRD), and Fourier-transform infrared spectroscopy (FTIR) were employed to elucidate the multi-scale synergistic enhancement mechanisms. The results indicate that hybrid modification significantly improves both static and dynamic performance. Specifically, the optimal hybrid proportion was identified as 1.5% NCC and 1.5 kg/m^3^ PPF. Under static conditions, this combined addition effectively increases compressive strength by 51.56% and enhances splitting tensile strength while also substantially improving material toughness. Under dynamic impact conditions, dynamic compressive strength is notably elevated by 62.47%, demonstrating a pronounced strain-rate strengthening effect. Microstructural analyses confirm the presence of a nano-densification and macro-crack bridging mechanism, wherein NCC chemically accelerates hydration and optimizes the cementitious matrix, thereby strengthening the fiber-matrix interfacial transition zone (ITZ). This robust ITZ maximizes the physical crack-bridging and energy dissipation capacities of the PPF network. Ultimately, this study provides critical experimental evidence and theoretical guidance for designing high-performance, impact-resistant concrete composites.

## 1. Introduction

The mechanical properties of concrete are vital for ensuring the safety of engineering structures. While concrete is recognized for its high static compressive strength, its inherent brittleness, low tensile capacity, and susceptibility to cracking significantly limit its applicability under high-strain-rate dynamic loadings, such as impacts and explosions [1,2,3]. In dynamic scenarios, concrete exhibits notably different mechanical responses and failure characteristics compared to static loading conditions. The principal limitations of traditional concrete, particularly its low energy absorption capacity, restrict its use in critical areas such as protective engineering and high-security infrastructure [4,5]. Furthermore, from a microstructural perspective, the presence of nano-scale pores significantly influences the macroscopic mechanical behavior and failure modes of the material [6,7]. Therefore, it is essential to enhance both the static and dynamic mechanical properties while optimizing the microstructural pore network to develop high-performance, impact-resistant concrete.

To address the defects of concrete, fiber reinforcement and nanomaterial modification have emerged as two effective strategies for enhancing toughness and reducing porosity. For instance, Wang [8] examined the effects of steel fibers, polyvinyl alcohol fibers, and basalt fibers on concrete strength, highlighting that the incorporation of these fibers significantly improved both the strength and toughness of the concrete. Extensive research shows that fibers can impede the propagation of internal microcracks and the formation of macroscopic cracks, thereby enhancing the tensile, flexural, impact, and fatigue resistance of concrete [9,10]. Polypropylene fiber (PPF) is widely utilized due to its low cost, excellent dispersibility, and stable chemical properties. It primarily creates a three-dimensional network structure within the concrete matrix, effectively bridging cracks and dissipating energy, which considerably enhances the toughness and crack resistance of the material [11,12]. Xu [13] investigated the impact of PPF content on concrete properties and found that an optimal PPF dosage (0.3–0.6%) produced a bridging effect, slowed the development of macroscopic cracks, increased ductility, and improved the compressive strength of the concrete. Moreover, a dosage of around 0.6% helped reduce internal pores. Similarly, Qi [14] found that an appropriate amount of PPF could significantly enhance concrete properties, noting that PPF bonds tightly with surrounding cementitious materials to prevent the development of internal microcracks. However, an excessive amount of fibers can lead to an increase in internal air voids, which may degrade the performance of the concrete. Additionally, driven by sustainable development, the use of recycled PP fibers in engineering has become increasingly widespread. To address performance degradation caused by thermo-oxidative aging during the recycling process, modifying recycled PP fibers with natural stabilizers, such as vitamin E and propyl gallate antioxidants, has emerged as a promising approach to enhance their mechanical and durability [15,16]. Using a split Hopkinson pressure bar (SHPB), Wei [17] analyzed the dynamic mechanical properties of PPF concrete, indicating that the PPF content has a significant impact on mechanical properties and exhibits a pronounced strain-rate dependency. As a novel inorganic nanomaterial, nano-calcium carbonate (NCC) not only enhances the strength and durability of cementitious composites but also improves their functional properties [18,19]. Cui [20] found that, in addition to accelerating the early hydration process of cement particles, NCC can improve the pore structure of gel composites. Li [21] observed a significant increase in hydration products in NCC concrete compared to ordinary concrete, noting that a 3% NCC dosage is optimal for enhancing long-term resistance to carbonation and chloride ion penetration. Wu [22] discovered that, beyond the physical mechanisms of nucleation and filling, NCC can react with tricalcium aluminate to form carboaluminates, thus reducing the porosity of the concrete. Additionally, while extensive research exists on the static mechanical properties of NCC concrete, studies on its dynamic mechanical properties are relatively scarce. Based on the intrinsic mechanisms of these two modification methods, researchers have started to investigate the synergistic effects of their combined application. They have found that nanomaterials can effectively address the limited improvements associated with single-fiber modification. For example, Chen [23] indicated that the hybrid addition of PPF and NCC can significantly enhance the mechanical properties of concrete, optimize its microstructure, and promote the hydration reaction. Additionally, Feng [24] discovered that NCC can enhance the surface roughness of PPF, which facilitates the formation of a high-density and highly hydrated C-S-H gel on the fiber surface, thereby improving the bonding strength with the cementitious matrix. In summary, although extensive research has been conducted on PPF-reinforced and NCC-modified concrete individually, there is still a notable gap in systematic investigations regarding the synergistic micro-mechanisms of their combined application under both static and dynamic loading conditions. This study posits that the nano-nucleation and physical filling effects of NCC can significantly densify the cementitious matrix and fundamentally enhance the interfacial transition zone (ITZ). This improved ITZ is expected to optimize the physical crack-bridging and pull-out energy dissipation capacities of PPF. Additionally, we propose that the synergistic mechanisms between the matrix and fibers may demonstrate distinct, rate-dependent responses when transitioning from static loading to high-strain-rate impacts.

This study employs compressive tests, splitting tensile tests, and split Hopkinson pressure bar (SHPB) tests to systematically evaluate hybrid-modified concrete. By designing various combinations of material dosages, we acquired the static and dynamic stress–strain responses of the composite, enabling a thorough analysis of the patterns of strength evolution and failure modes. Additionally, through the use of scanning electron microscopy (SEM), X-ray diffraction (XRD), and Fourier-transform infrared spectroscopy (FTIR), we examined the microstructural and phase composition changes to further clarify the underlying micro-mechanisms influencing macroscopic mechanical responses. Ultimately, from both macroscopic and microscopic viewpoints, this study seeks to elucidate the synergistic enhancement mechanisms of the two modifying materials under high-strain-rate impact conditions, thereby establishing a solid theoretical foundation for developing high-performance, impact-resistant concrete.

## 2. Experimental Materials and Methods

### 2.1. Experimental Materials and Mix Proportions

**Figure 1:**
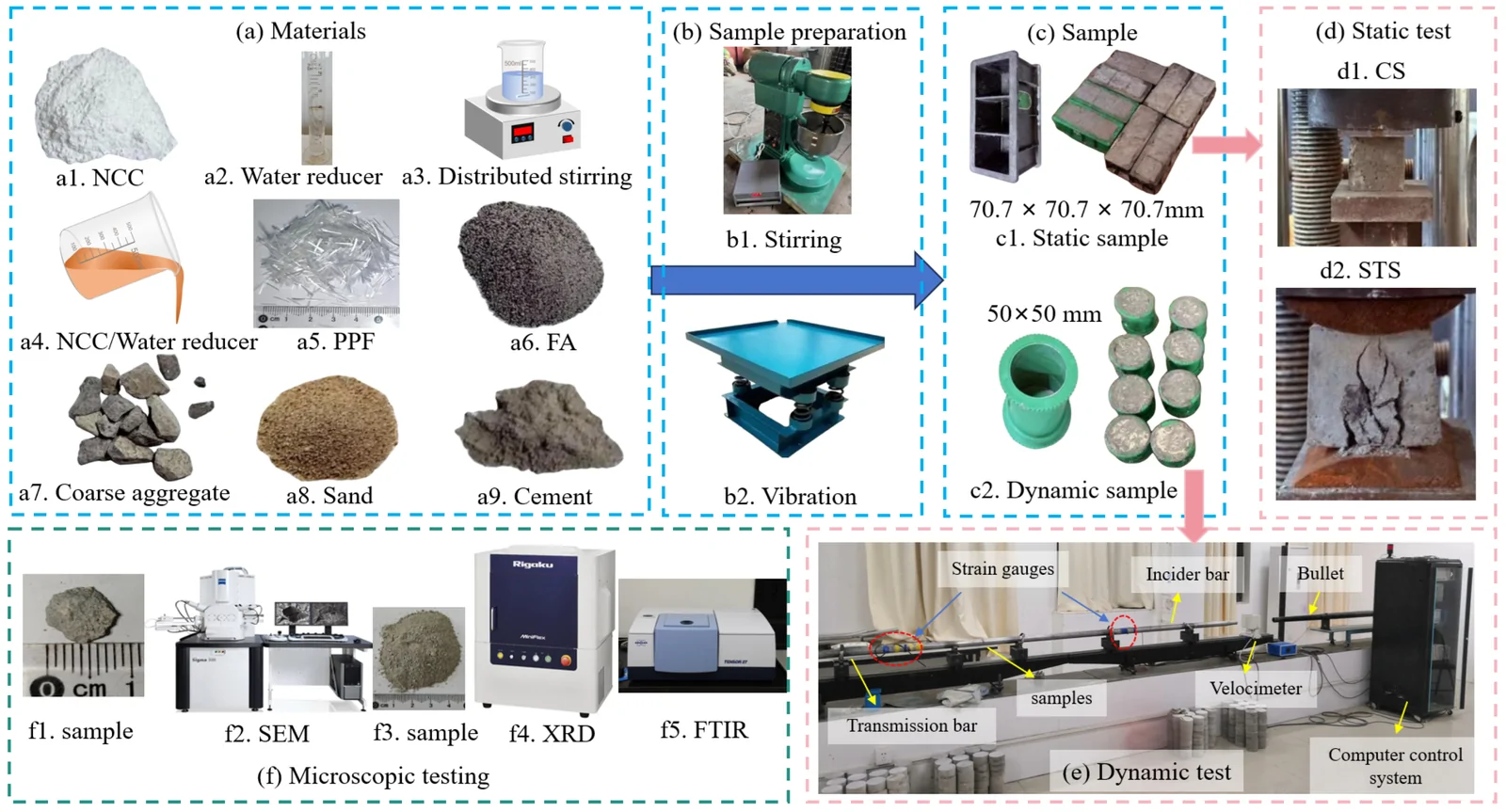
NCC/PPF Concrete Preparation and Testing Procedure.

As shown in Figure 1a, the cement used in the experiment was Bagongshan P·O 42.5 ordinary Portland cement sourced from Anhui, China. It has a specific surface area (fineness) of 345 m^2^/kg, and its standard compressive and flexural strengths at 28 days are 48.5 MPa and 7.8 MPa, respectively. The chemical composition is detailed in Table 1. The control concrete formulation, designated as NP-0, was meticulously designed to serve as a moderate-strength baseline. By incorporating 75 kg/m^3^ of fly ash, the early-age hydration strength was effectively regulated. This design aims to achieve a balance between the matrix bond strength and the tensile strength of the polypropylene fibers (PPF), ensuring that the flexible fibers experience a pull-out and energy dissipation process rather than premature rupture during crack propagation. This aspect is crucial for assessing the synergistic impact resistance. The fly ash was sourced from the stockyard of the Shangyao Power Plant in Huainan (Anhui, China), primarily consisting of SiO_2_ (52.5%) and Al_2_O_3_ (28.0%). River sand with a fineness modulus of 2.6 was selected as the fine aggregate, and a liquid polycarboxylate superplasticizer was used as the water-reducing agent. Crushed stone with a nominal particle size ranging from 5 to 20 mm was utilized as the coarse aggregate. The PPF used in this study was 100% pure chopped polypropylene fiber, sourced from Changsha (Hunan, China), with lengths of 9 mm and 12 mm. To avoid complex geometric variables, straight monofilament fibers were specifically chosen to accurately investigate the NCC-PPF synergistic mechanism, which focuses on how NCC densifies the interfacial transition zone (ITZ) to improve the pull-out resistance of the fibers. The specific parameters are outlined in Table 2. The NCC utilized in the tests was manufactured by Shanghai Chenqi Chemical Technology Co., Ltd. (Shanghai, China), with the specific material parameters detailed in Table 3.

In this study, NP-0 concrete was selected as the control group. The mix proportion was derived from the formulation proposed by Yu et al. [25] and further optimized to ensure adequate workability. The composition per cubic meter of concrete is as follows: 461 kg of cement, 75 kg of fly ash, 175 kg of water, 512 kg of fine aggregate, 1252 kg of coarse aggregate, and 2.5 kg of superplasticizer (Table 4). Based on this baseline mix proportion, the total quantity of materials was maintained while the dosages of nano-calcium carbonate (NCC) and polypropylene fiber (PPF) were varied. The specific naming conventions for the experiments are detailed in Table 5. The NCC dosage was included as a mass percentage of the total cementitious materials, while the PPF dosage was calculated by mass based on its volume percentage (kg/m^3^).

### 2.2. Sample Preparation

As illustrated in Figure 1a–c, nano-calcium carbonate (NCC) was pretreated using a superplasticizer-assisted ultrasonic dispersion method prior to specimen preparation to prevent agglomeration. First, the NCC powder and polycarboxylate superplasticizer were manually stirred to form a paste-like premix. This premix was then gradually added to the mixing water and magnetically stirred at 500 rpm for 15 min to achieve preliminary dispersion. Following this, ultrasonic dispersion was applied at a frequency of 40 kHz for 25 min, after which the mixture was allowed to stand and age for 50 min. Mixing was conducted using a compulsory concrete mixer according to the specified mix proportions. Aggregates were loaded first, followed by cement and fly ash in sequence. The aggregates and cementitious materials were dry-mixed for about 2 min to ensure uniform blending. The NCC dispersion and water were then added simultaneously. After 1 min of mixing, fibers were sifted into the mixture while continuous stirring was maintained to ensure their uniform distribution. Mixing continued for an additional 2 min before the mixer was turned off. The concrete was subsequently discharged, cast into molds, and demolded after 24 h. The specimens were then cured in a standard environment at 20 °C and 95% relative humidity until they reached the designated test age of 28 days. Compressive strength tests and splitting tensile tests utilized 70.7 mm × 70.7 mm × 70.7 mm cube specimens, while the SHPB tests employed 50 mm × 50 mm cylindrical specimens. The specific testing equipment and specimens are illustrated in Figure 1b,c.

### 2.3. Mechanical Testing

#### 2.3.1. Static Mechanical Testing

(a) Compressive strength Test

As illustrated in Figure 1d, the tests were conducted following the Standard for Test Methods of Concrete Physical and Mechanical Properties (GB/T 50081-2019) [26]. Prior to testing, the actual dimensions of the specimens were measured with a high-precision caliper, and their surfaces were wiped with a cloth to ensure proper contact with the bearing plates of the testing machine. A microcomputer-controlled electro-hydraulic servo universal testing machine (SINOTEST, csc-yaw3000, Changchun, China) was utilized to apply a continuous load at a constant rate of 1 mm/min until the specimens completely failed. Four replicate specimens were tested for each experimental group.

(b) Splitting tensile Strength Test

As illustrated in Figure 1d, the tests were conducted in accordance with the Standard for Test Methods of Concrete Physical and Mechanical Properties. Prior to testing, a steel bearing strip, measuring 15 mm in width and 4 mm in thickness, was placed between the upper and lower bearing surfaces of the specimen and the loading plates. This configuration ensured that the load was applied as a line load along the specimen’s diametrical direction. The same microcomputer-controlled electro-hydraulic servo universal testing machine, which was used for the unconfined compressive strength tests, was employed to apply a continuous load at a constant rate of 1 mm/min until the specimens failed completely. Three replicate specimens were tested for each experimental group.

#### 2.3.2. SHPB Test

The SHPB technique, as shown in Figure 1e, was employed to assess the dynamic compressive properties of concrete specimens using an impact gas pressure of 0.3 MPa. The apparatus consists of a launching system, a velocity measurement system, a bar system (which includes an incident bar and a transmitted bar), and a data acquisition system. For each material group, at least four valid replicate tests were conducted at each impact velocity. The parallelism error of the specimen end faces was kept below 0.02 mm to ensure compliance with the one-dimensional stress assumption of the SHPB test. Furthermore, to guarantee that the specimens achieved stress equilibrium prior to failure and to obtain a smooth incident waveform, a circular copper disk with a diameter of 8 mm and a thickness of 1 mm was affixed to the end of the striker bar as a pulse shaper in all tests.

Driven by high-pressure gas, the striker bar impacts the incident bar, generating an incident stress pulse (*ε_I_*). As this pulse propagates to the bar-specimen interface, a portion is reflected, resulting in a reflected wave (*ε_R_*), while the remainder transmits through the specimen, forming a transmitted wave (*ε_T_*). Strain gauges attached to the bars record these three key signals. Using one-dimensional stress wave theory, the stress–strain relationship of the specimen can be calculated via the three-wave method, as illustrated in Equations (1)–(3) [27,28].

(1) $$\overset{\cdot }{\varepsilon }\left(t\right)=\frac{c_{0}}{L_{s}}\left[\varepsilon_{I}\left(t\right)-\varepsilon_{R}\left(t\right)-\varepsilon_{T}\left(t\right)\right]$$

(2) $$\varepsilon \left(t\right)=\frac{c_{0}}{L_{s}}\int_{0}^{t}\left[\varepsilon_{I}\left(t\right)-\varepsilon_{R}\left(t\right)-\varepsilon_{T}\left(t\right)\right]\mathrm{dt}$$

(3) $$\sigma \left(t\right)=\frac{S_{B}E}{S_{S}}\left(t\right)$$

where $\overset{\cdot }{\varepsilon }\left(t\right)$, *e*(*t*), and *s*(*t*) are the strain rate, strain, and stress of the specimen, respectively; *c*_0_, *L_s_*, *S_B_*, *S_S_*, *E* represent the elastic compressive wave velocity, the length of the specimen, the cross-sectional area of the bars, the cross-sectional area of the specimen, and the elastic modulus of the bars, respectively; *e_I_*(*t*), *e_R_*(*t*), and *e_T_*(*t*) denote the collected incident, reflected, and transmitted pulse strains, respectively.

### 2.4. Microscopic Test

A Bruker D8 ADVANCE X-ray diffractometer (Bruker, Ettlingen, Germany) was utilised to conduct a qualitative analysis of the hydration products after 28 days of standard curing. Before testing, the targeted paste samples were immersed in absolute ethanol for 24 h to halt the hydration process and were subsequently dried in an oven at 60 °C for 24 h. The paste specimens were then ground and sieved using a 45 μm round-hole sieve. For the X-ray diffraction (XRD) testing, the scanning angle range was set from 5° to 70° with a scanning rate of 2°/min, as shown in Figure 1f.

Samples extracted from the fractured regions underwent scanning electron microscope (SEM) testing to observe the morphology of the fibres and the matrix following concrete failure under various conditions. The dimensions of the samples were approximately 7 mm × 7 mm × 7 mm. Prior to testing, the samples were dried in an oven at 60 °C for 24 h. A Sigma 360 high-resolution field emission scanning electron microscope (Zeiss, Oberkochen, Germany) was employed to acquire secondary electron images. To improve electrical conductivity, the samples were sputter-coated with gold before observation, as illustrated in Figure 1f.

Fourier-transform infrared spectroscopy (FTIR) was used to analyse the trend of changes in major functional groups. FTIR measurements were conducted using a Nicolet iS5 infrared spectrometer (Thermo Fisher Scientific, Waltham, MA, USA) employing the KBr pellet method. A mixture of 1 mg of dried powder sample and 100 mg of KBr powder was uniformly blended and pressed into pellets. During the test, both the sample and background scan numbers were set to 32, with the acquisition range defined from 4000 to 400 cm^−1^. The specimen preparation procedure was consistent with that of the XRD tests, as shown in Figure 1f.

## 3. Test Results and Analysis

### 3.1. Static Mechanical Test Results

#### 3.1.1. Compressive Strength Test

(a) Effect of Single-Factor Modification on Compressive Strength

**Figure 2:**
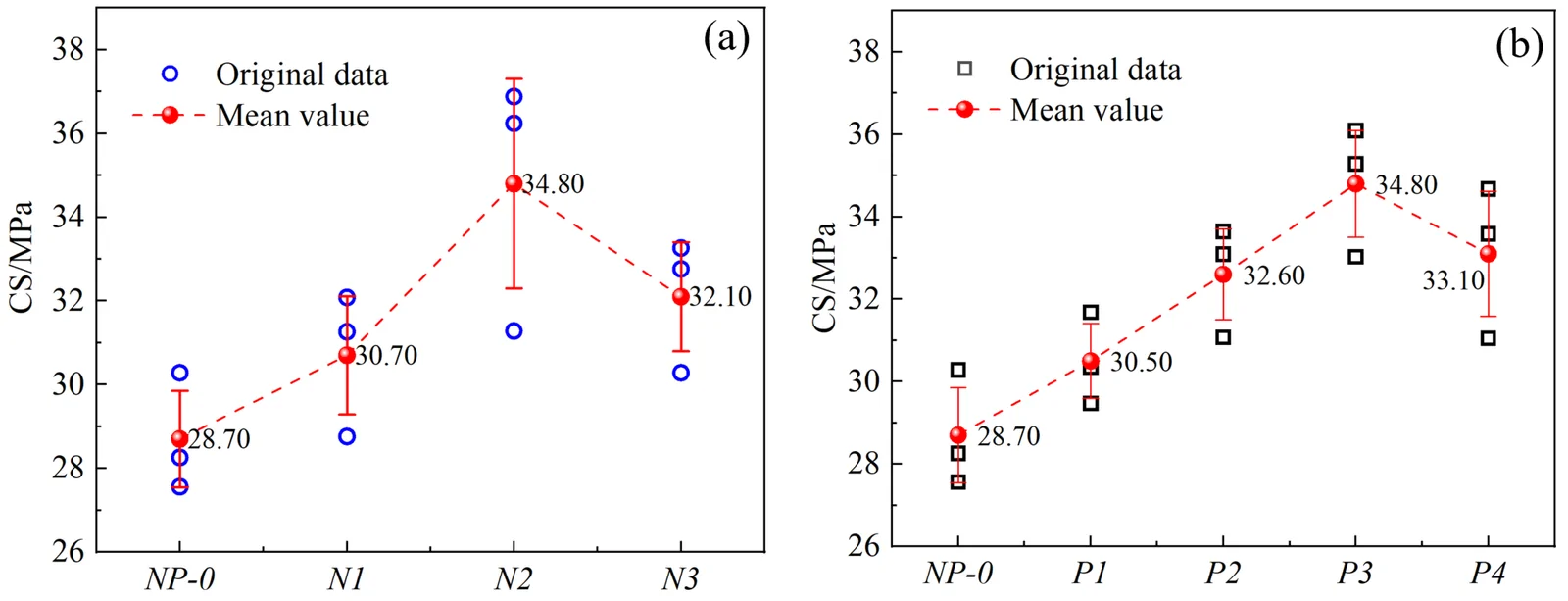
The influence of NCC and PPF single doping on the strength of concrete. (**a**) NCC concrete, (**b**) PPF concrete.

As illustrated in Figure 2, the variation in the static compressive strength of concrete modified with single components, NCC and PPF, is presented. The compressive strength (CS) of the control group, NP-0, was measured at 28.70 MPa. Notably, the 28-day compressive strength of standard 100 mm cubes in Yu et al. [25] was 21.14 MPa, whereas the compressive strength in this study reached 28.70 MPa. This apparent strength enhancement is attributed to the classical statistical size effect of Weibull theory [29]. An increase in the NCC dosage resulted in enhanced compressive strength, peaking at 34.80 MPa with a dosage of 1.5%, which corresponds to an approximate increase of 21.25% compared to NP-0. However, as the dosage was further increased, the compressive strength decreased to 32.10 MPa. Existing research indicates that incorporating nanomaterials into concrete leads to a combined effect of micro-aggregation, crystal nucleation, and filling, which accelerates cement hydration, improves matrix densification, and enhances the overall performance of the matrix [16]. For specimens modified with single-component PPF, the compressive strength initially increased with the PPF dosage, reached a peak, and then decreased. At a PPF dosage of 1.5 kg/m^3^, the compressive strength of the specimens also peaked at 34.80 MPa, reflecting a 21.25% increase compared to the control group. These experimental results suggest that both NCC and PPF effectively enhance the compressive strength of concrete. Furthermore, the compressive strength of specimens modified with either NCC or PPF exhibits a rise-then-fall trend as the dosage increases, indicating an optimal dosage for both materials. Some studies have noted that excessive NCC particles can agglomerate due to dispersion issues, negatively impacting matrix densification [30], while exceeding a certain threshold of PPF can hinder effective fiber dispersion, leading to a reduction in strength [31].

(b) The Effect of NCC/PPF Hybrid Incorporation on the Compressive Strength of Concrete

**Figure 3:**
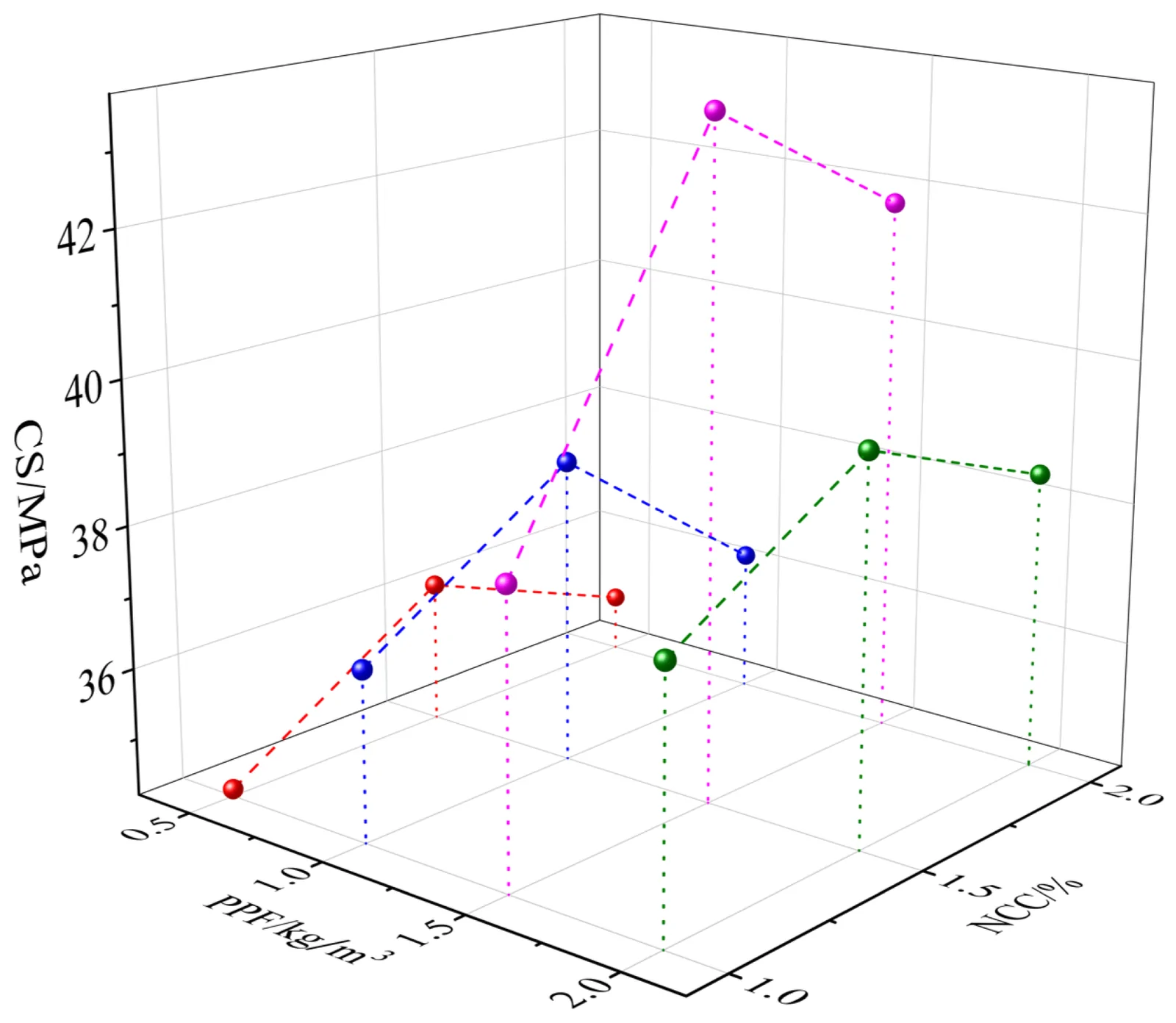
The influence of NCC and PPF admixtures combined use on the strength of concrete.

Figure 3 illustrates the variation in static compressive strength of Nano-calcium carbonate/Polypropylene Fiber Concrete (NPC). Compared to the control group NP-0, which exhibited a compressive strength (CS) of 28.70 MPa, all NPC specimens showed varying degrees of improvement in CS. When the dosage of Polypropylene Fiber (PPF) was set at 1.5 kg/m^3^ and the dosage of Nano-calcium Carbonate (NCC) at 1.5%, the static compressive strength of the NPC peaked at approximately 43.5 MPa. This result indicates a significant positive synergistic enhancement mechanism between the nanomaterial NCC and the PPF fibers. With the PPF dosage maintained at 1.5 kg/m^3^, increasing the NCC dosage from 1.0% to 1.5% resulted in a CS increase from approximately 37.5 MPa to 43.5 MPa, an absolute increase of 6.0 MPa, demonstrating a steep strength growth gradient. Conversely, when the NCC dosage was fixed at 1.5%, increasing the PPF dosage from 1.0 kg/m^3^ to 1.5 kg/m^3^ led to a CS increase of about 4.5 MPa (from roughly 39.0 MPa to 43.5 MPa). These findings suggest that under static compressive loading conditions, concrete strength is more sensitive to changes in NCC dosage; thus, NCC plays a larger and more dominant role in enhancing compressive strength. The underlying mechanical basis for this phenomenon is that the uniaxial compressive strength of concrete fundamentally depends on the initial densification of the solid matrix and the number of micro-defects present. NCC directly influences the hydration process of the cement matrix and optimizes the pore structure, resulting in increased compressive strength. In contrast, PPF, as a polymer fiber, primarily contributes to crack bridging and restricts lateral deformation during the later stages of loading. Consequently, its direct impact on static ultimate compressive strength is less significant than the densification effects provided by the nanomaterial.

Furthermore, Figure 3 illustrates the threshold effect of the modified materials. When the dosage of these materials exceeded the optimal value (i.e., reaching the 2.0% level), a decline in macroscopic strength was observed across all cases. Specifically, an increase in the NCC dosage to 2.0% led to a decrease in strength to approximately 42.5 MPa. This reduction occurs because an excess of nanoparticles, which possess a large specific surface area and extremely high surface energy, is highly prone to agglomeration within the matrix. These agglomerates fail to effectively perform the functions of filling and nucleation; instead, they adsorb a significant amount of free water, resulting in incomplete local hydration and the formation of micro-scale defects. Conversely, when the PPF dosage reached 2.0 kg/m^3^, the strength ranged between 36.5 and 39.5 MPa. High dosages of flexible fibers are particularly vulnerable to entanglement and fiber balling during the mixing process, which not only disrupts the continuity of the concrete matrix but also introduces numerous interconnected harmful air voids within the clusters, ultimately leading to a reduction in strength [32].

(c) The Effects of NCC and PPF on the Stress–Strain Behavior of Concrete

**Figure 4:**
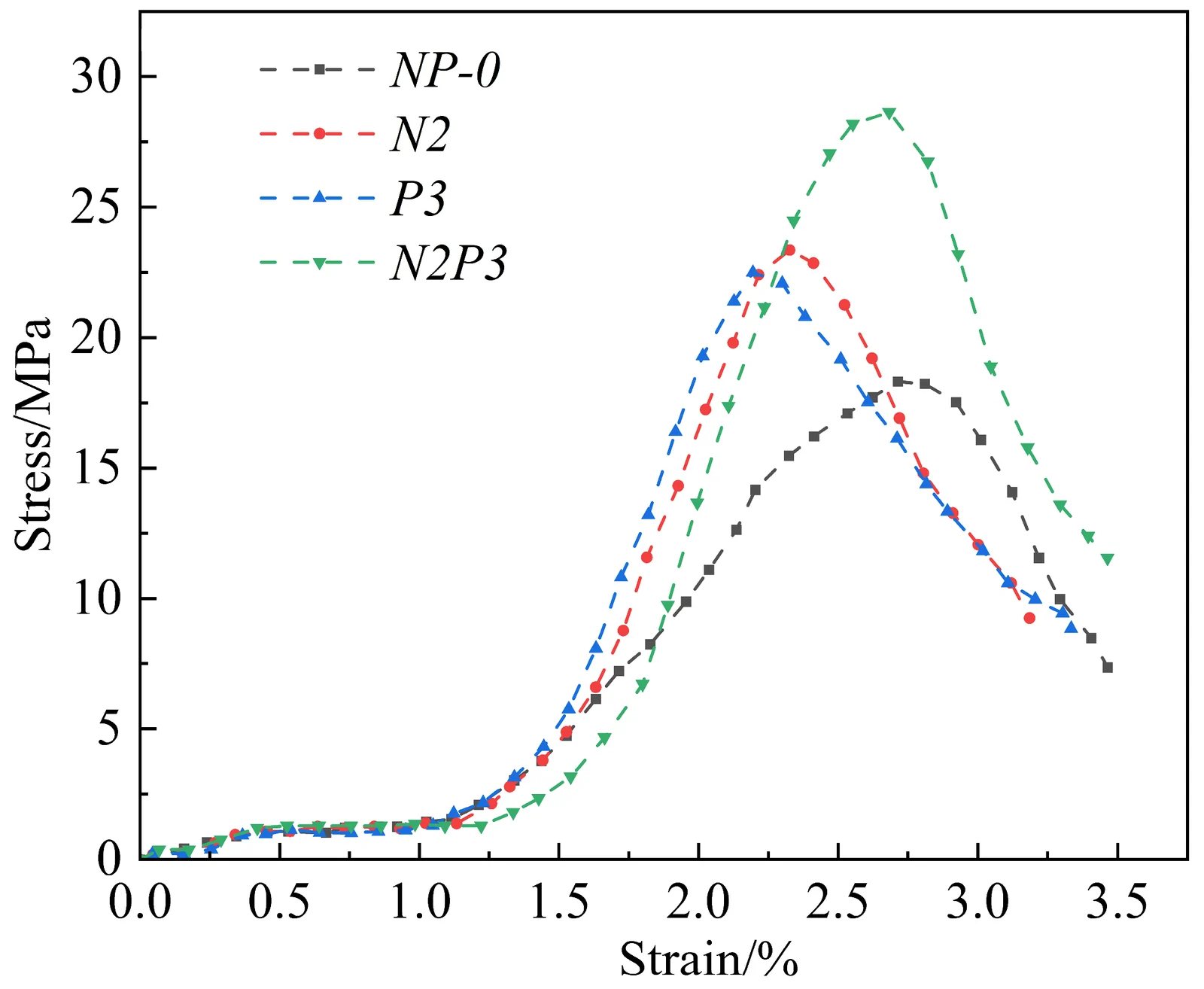
Typical static stress–strain relationship curve of NCC/PPF concrete.

As indicated by the stress–strain curves presented in Figure 4, the linear elastic stages of the N2 and P3 curves are significantly steeper than those of the control group NP-0. This suggests that the incorporation of NCC and PPF as single components enhances the initial elastic modulus and stiffness of the concrete. Additionally, the peak strains at peak stress for N2 and P3 are 2.1% and 2.3%, respectively, which are notably lower than the 2.7% peak strain observed in the control group NP-0. This indicates that while single-component modifications with NCC or PPF improve strength and stiffness, they also lead to a reduction in the material’s ability to withstand deformation before failure, resulting in increased brittleness. In contrast, the peak strain for the N2P3 combination is 2.6%, suggesting that the enhancement in strength comes with a mitigation of the toughness reduction typically associated with single-component modifications, allowing for greater deformation prior to failure. Beyond the peak stress, the curves transition into the post-peak softening stage, where the N2P3 curve exhibits the largest integral area. Since the area under the stress–strain curve represents the strain energy of the material [27], this finding indicates that the N2P3 combination possesses optimal energy dissipation capacity and toughness. Furthermore, the N2P3 curve not only maintains a higher slope but also retains the linear characteristics of the elastic stage for an extended duration, demonstrating that the hybrid incorporation of NCC and PPF significantly enhances the overall elastic bearing capacity of the material while providing the specimens with superior ductility.

#### 3.1.2. The Results of Splitting Strength Test

**Figure 5:**
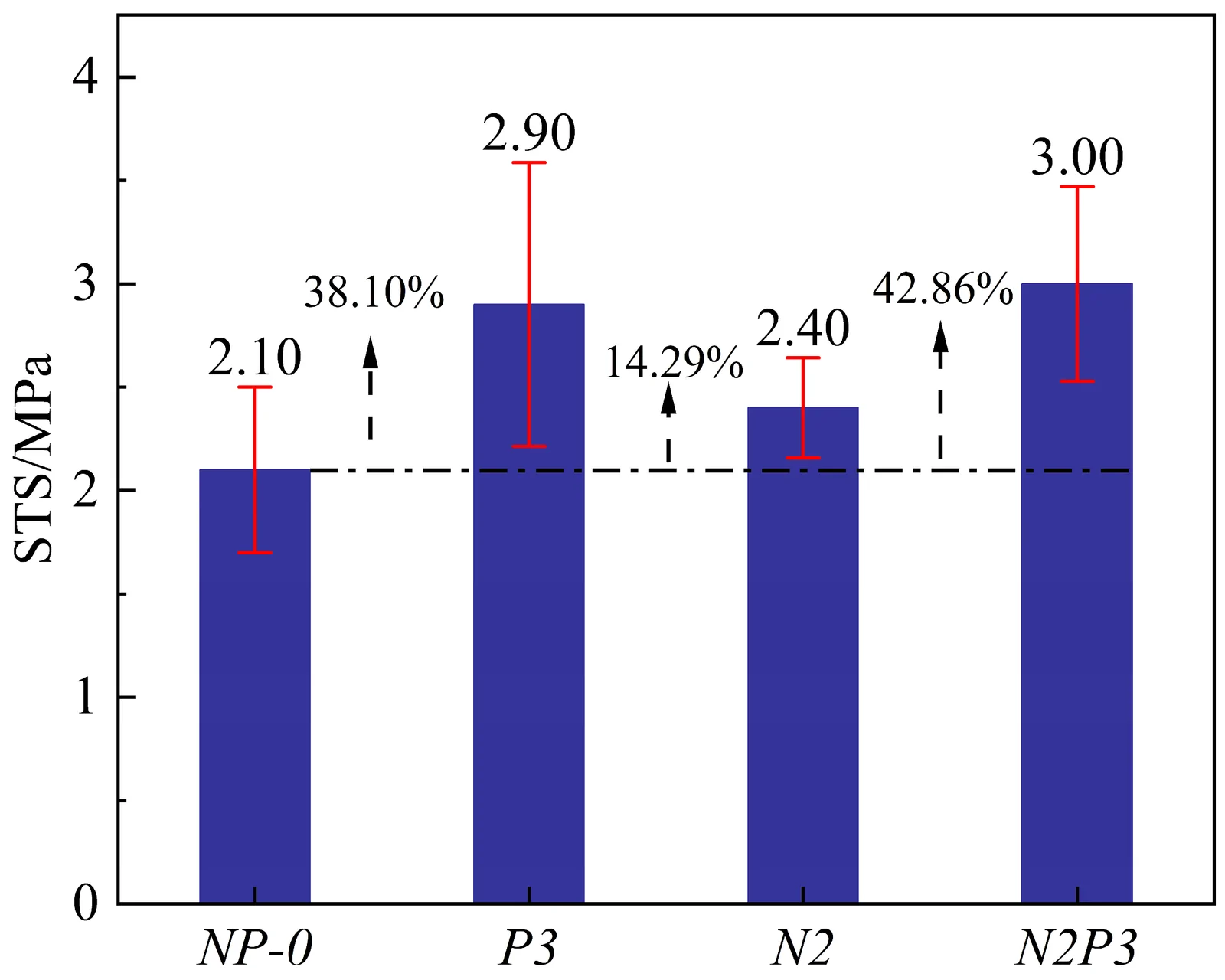
Typical NCC/PPF concrete splitting tensile strength.

Figure 5 presents the results of the splitting tensile strength (STS) tests conducted on specimens from the control group, as well as those modified with individual components and a hybrid optimal mix proportion. The STS for the control group, identified as NP-0, was measured at just 2.10 MPa. For the single-component modification using NCC (N2), the STS increased to 2.40 MPa, reflecting a 14.29% enhancement. This suggests that NCC’s filling effect positively influences the tensile strength of the concrete. In contrast, the single-component modification with PPF (P3) resulted in a more pronounced increase in STS, reaching 2.90 MPa, which represents a 38.10% rise. This improvement is significantly greater than that achieved with NCC, indicating that PPF effectively provides a bridging effect within the concrete, thereby enhancing the splitting tensile strength. The hybrid modification combining N2 and P3 (N2P3) resulted in an STS peak of 3.00 MPa, a notable increase of 42.86% compared to NP-0. These findings demonstrate a beneficial synergistic effect between the two materials, significantly enhancing the tensile performance of the concrete.

#### 3.1.3. Sample Failure Mode

**Figure 6:**
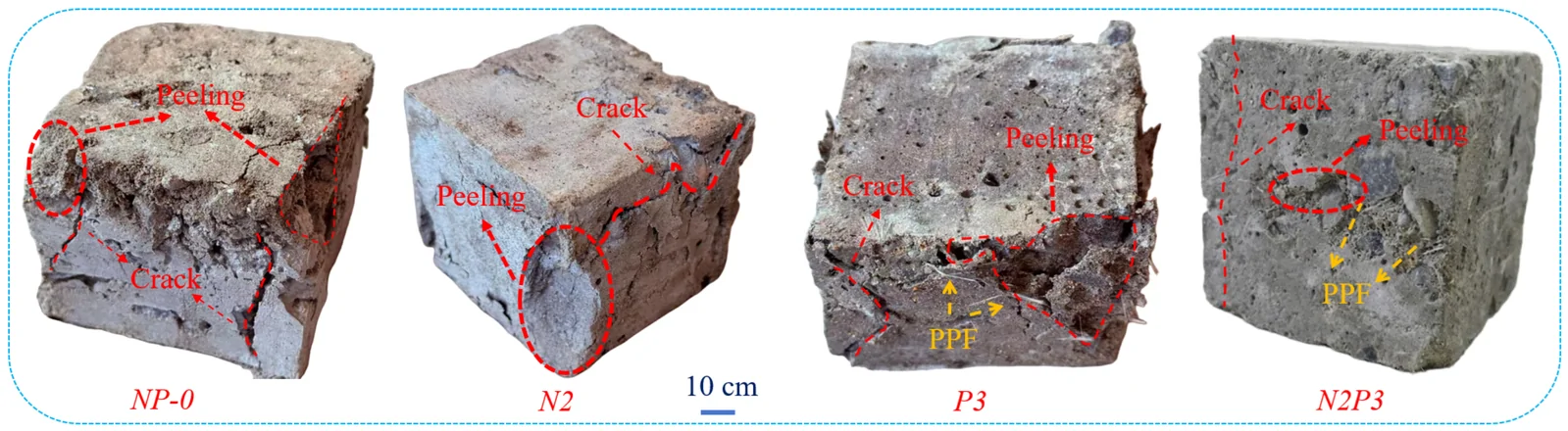
Typical NCC/PPF concrete failure mode.

As illustrated in Figure 6, the failure images of the specimens subjected to compressive strength tests are presented. The control group, NP-0, exhibited typical brittle failure characteristics. In the later stages of loading, cracks propagated rapidly, and the concrete cover surrounding the specimens experienced extensive spalling, ultimately resulting in a standard inverted-cone failure mode. The failure mode of the specimens modified with NCC remained primarily brittle; however, compared to the control group, the spalled fragments were potentially larger or featured sharper edges. This suggests that while NCC enhances the internal densification and ultimate compressive strength of the matrix, it does not fundamentally alter the brittle nature of the matrix, which tends to fail quickly upon cracking. In contrast, the specimens modified with single-component PPF displayed a notable shift toward ductile failure. The fibers at the surfaces where the concrete cover spalled or where corners broke off demonstrated a connecting effect, showcasing superior compressive integrity along with high strength and toughness. Furthermore, the failure of the NP-0 and N2 specimens was characterized by a few prominent through-going macroscopic cracks. Once these main cracks formed, stress was released rapidly, leading to the development of wide cracks and local spalling of the concrete. However, in the P3 and N2P3 specimens, the propagation of main cracks was significantly inhibited, resulting in a dense network of multiple cracks. This is attributed to the three-dimensional network created by the PPF within the matrix, which altered the stress transmission paths. Consequently, stress that was originally concentrated in one area became dispersed, transforming large cracks into numerous fine cracks and significantly enhancing the material’s energy dissipation capacity during the failure process.

**Figure 7:**
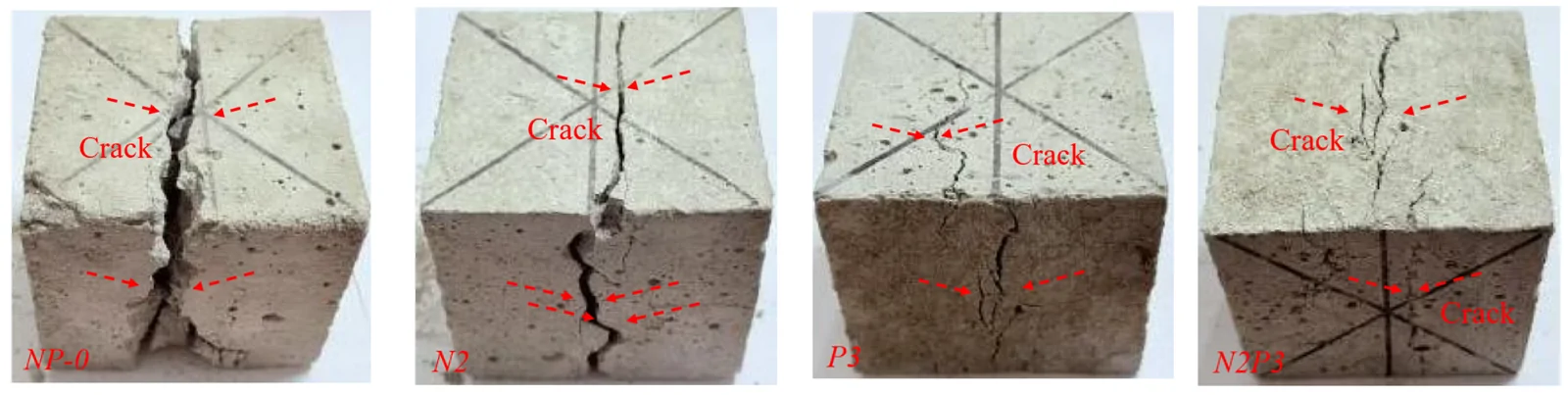
Typical cracking failure mode of NCC/PPF concrete.

As illustrated in Figure 7, the NP-0 specimen exhibited a typical brittle cleavage failure. Upon reaching the ultimate splitting tensile stress, the specimen split instantaneously along the loading axis into two halves. The main crack was notably wide and propagated through the entire cross-section, with the fracture surface remaining relatively smooth, indicating very low fracture energy dissipation and poor post-failure integrity. Similarly, the failure mode of the N2 specimen was predominantly brittle, characterised by a single macroscopic crack. This suggests that, while the nano-filling and nucleation effects of NCC effectively enhanced the densification and compressive hardness of the matrix and possibly increased the initial cracking tensile stress threshold, the nanoparticles were insufficient to provide the tensile bonding force necessary to bridge macroscopic cracks. Once micro-cracks coalesced into a macroscopic crack, the stress was released rapidly, leading to instantaneous instability and fracture of the matrix. This implies that relying solely on nano-scale densification cannot fundamentally address the inherent defect of concrete’s susceptibility to brittle fracture under tension. In contrast, the failure mode of the P3 specimen demonstrated a significant change, exhibiting excellent ductile failure characteristics, where the specimen was cracked but not broken. Although splitting cracks appeared on the surface along the loading direction, the crack width was significantly limited, allowing the specimen to maintain a good overall geometric configuration after failure without complete separation. This phenomenon illustrates the bridging effect of polypropylene fibres (PPF). When the matrix cracked, the flexible polypropylene fibres spanning the crack sides acted like micro-tie rods, effectively bearing and transferring tensile stress across the crack. This significantly increased resistance to crack propagation and transformed the originally concentrated fracture energy into dissipative energy during the processes of fibre pull-out and rupture. The N2P3 specimen demonstrated the highest tensile cross-sectional integrity. In comparison to the single-component PPF group, the main crack in the hybrid group was extremely fine, exhibiting characteristics of micro-cracking. This experimental outcome suggests that, within the hybrid system, the hydration-promoting effect of NCC generated denser C-S-H gel on the surface of the PPF, effectively eliminating interfacial pore defects that could easily arise from single-component fibre modification. This, in turn, provided a robust anchoring matrix for the PPF. When subjected to tensile cracking, the enhanced interfacial bonding force made it more challenging for the PPF to be easily pulled out, compelling the fibres to exert higher tensile strength. Ultimately, this achieved a perfect synergy between the NCC-strengthened anchoring matrix and the PPF’s cross-crack bridging and energy-dissipating capabilities, transforming the macroscopic main crack into multiple dispersed, fine micro-cracks.

### 3.2. Results of Dynamic Mechanical Tests

#### 3.2.1. Results of Dynamic Compressive Strength Tests

**Figure 8:**
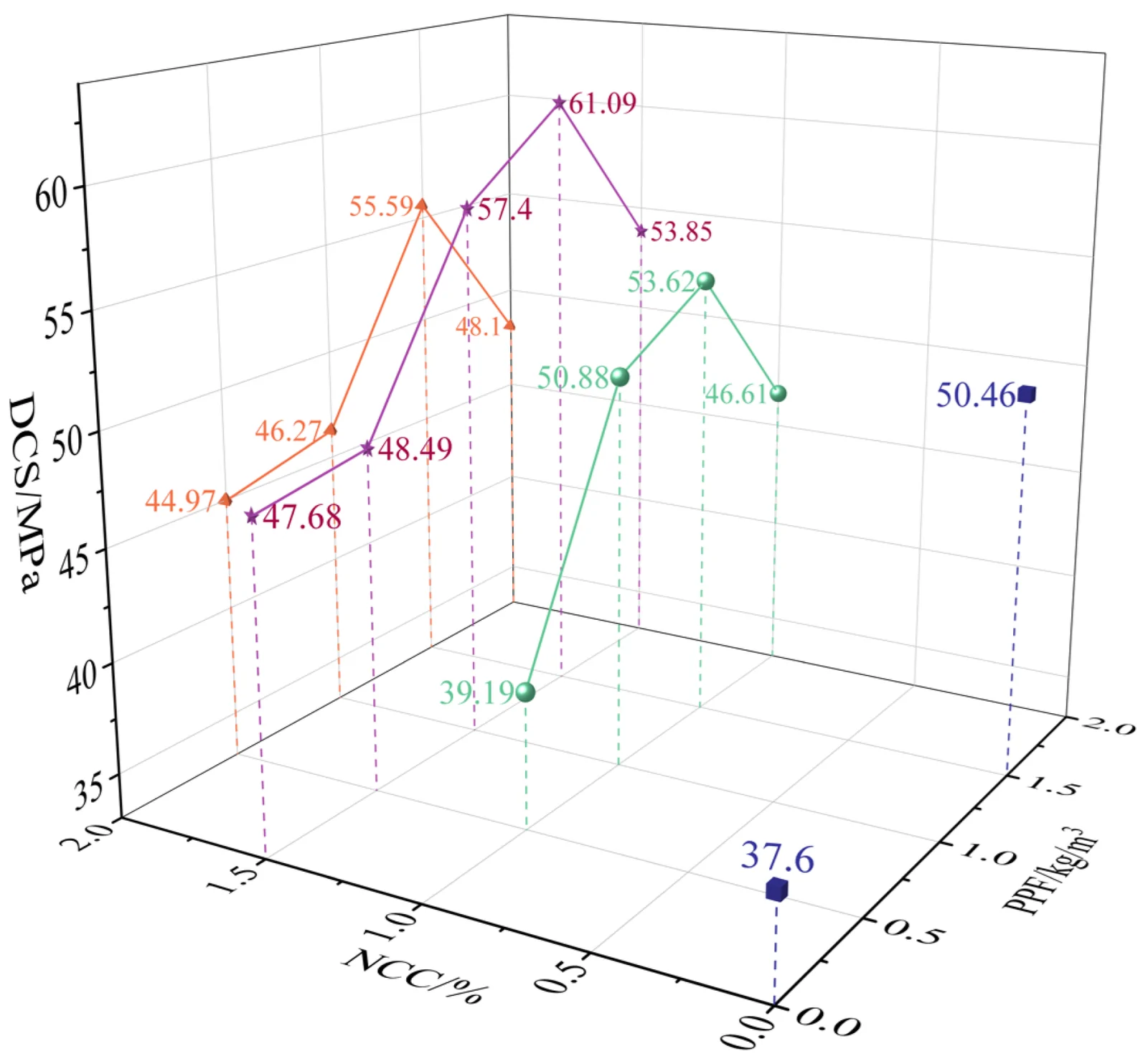
NCC/PPF concrete dynamic compressive strength.

The dynamic compressive strength (DCS) under various hybrid mix proportions of NCC and PPF exhibits significant non-linear spatial distribution characteristics, as shown in Figure 8. It reaches a global peak of 61.09 MPa when both the NCC and PPF dosages are set at 1.5% and 1.5 kg/m^3^, respectively. Compared to the NP-0 control group, which recorded a DCS of 37.6 MPa, this represents a notable increase of 62.47%. These experimental results demonstrate a cross-scale synergistic enhancement effect in resisting impact loads. During the extremely short duration of the impact wave, the pores refined by NCC and the enhanced interfacial transition zone (ITZ) effectively slow down the transmission of initial stress waves and the formation of micro-cracks. Simultaneously, the three-dimensional network formed by PPF provides significant bridging resistance at the moment of macroscopic crack formation. Moreover, the dynamic test results reveal a pattern of shifting dominance. Within the optimal proportion range, fixing the NCC dosage at 1.5% while increasing the PPF dosage from 0.5 kg/m^3^ to 1.5 kg/m^3^ results in a substantial increase in DCS of 13.41 MPa. Conversely, when the PPF dosage is fixed at 1.5%, raising the NCC dosage from 1.0% to 1.5% results in a smaller increase of only 7.47 MPa. This suggests that, under dynamic high-strain-rate impact, the influence of PPF on strength surpasses that of NCC and holds a dominant position. Under impact loading, micro-cracks within the matrix exhibit characteristics of multi-point concurrency and rapid propagation. Relying solely on NCC for improved matrix densification provides limited energy absorption, while energy dissipation through the physical bridging, pull-out, and rupture of PPF serves as the primary mechanism for resisting dynamic failure. Furthermore, the dynamic strength demonstrates a notable threshold degradation effect. When the dosage of NCC or PPF is increased to 2.0%, a significant decline in DCS is observed. This decline is attributed to micro-defects introduced by excessive dosages, such as fissures caused by NCC or PPF agglomeration, which are exacerbated by inertial effects, leading to a reduction in specimen strength under impact loading.

#### 3.2.2. Typical Stress–Strain Curves of Specimen

**Figure 9:**
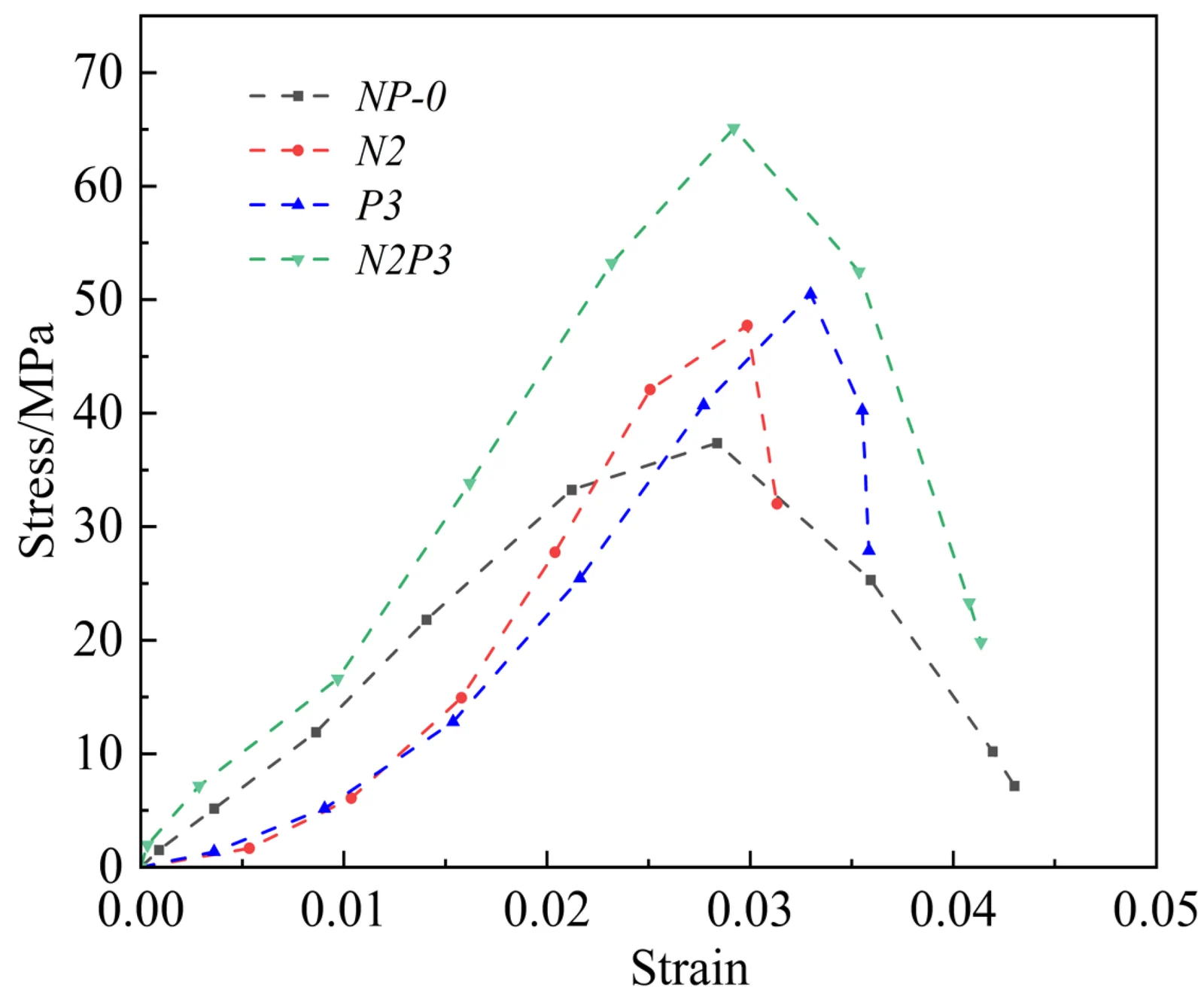
Typical dynamic stress–strain relationship curve of NCC/PPF concrete.

Figure 9 displays the typical dynamic stress–strain curves for the specimens NP-0, N2, P3, and N2P3, illustrating the deformation history of the materials subjected to high strain rates. These curves can be divided into three distinct stages: an elastic ascending stage, a plastic yielding stage, and a post-peak softening stage. Regarding the initial elastic modulus, represented by the slope of the ascending stage, the groups containing NCC (N2 and N2P3) show significantly steeper slopes compared to the control group (NP-0) and the single-component PPF group (P3). This suggests that the nano-densification effect of NCC effectively enhances the dynamic stiffness of the matrix. Furthermore, when analyzing peak strain and ductility characteristics, the peak point of the single-component PPF group (P3) shifts notably to the right, indicating an increase in peak strain, while the post-peak descending stage becomes more gradual. These experimental results demonstrate that the bridging effect of PPF enables the concrete to achieve superior deformation capacity and crack resistance. Ultimately, the optimal hybrid group (N2P3) has the largest curve envelope area, which signifies dynamic energy absorption capacity and toughness. This group not only achieves the highest peak stress but also retains excellent deformation capacity, highlighting the synergistic and complementary mechanisms of NCC in enhancing stiffness and strength, as well as PPF in improving ductility and energy dissipation.

**Figure 10:**
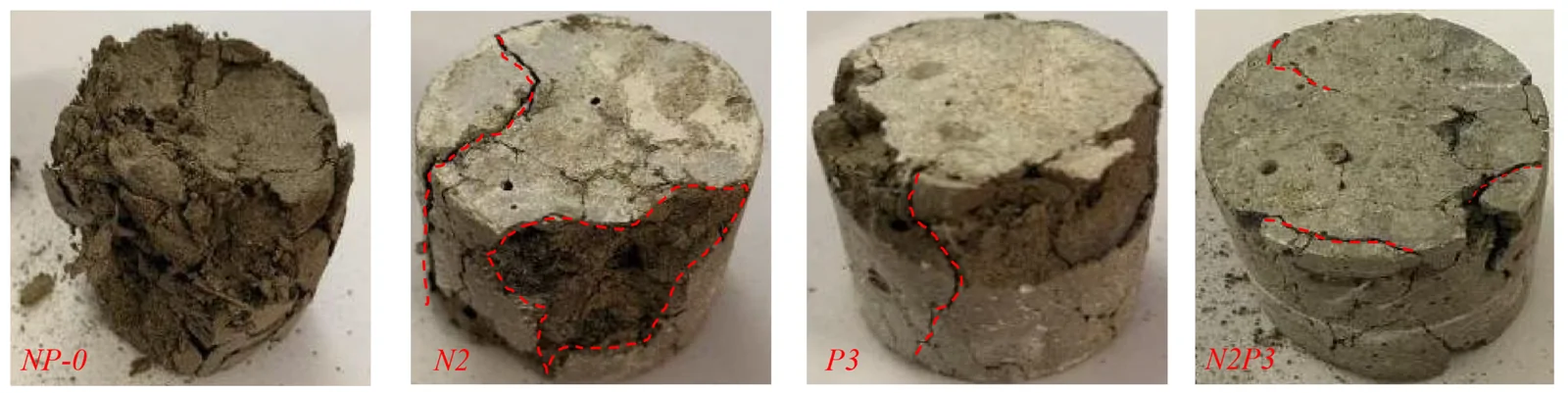
Typical failure mode of NCC/PPF concrete under impact load.

Figure 10 presents the failure images of typical specimens (NP-0, N2, P3, and N2P3). The dynamic failure characteristics indicate a notable transition from brittle pulverization to high-toughness energy absorption. The control group NP-0 exhibited typical pulverization failure. Under the intense impact of transient high-frequency stress waves, micro-cracks within the brittle matrix propagated rapidly, leading to a complete loss of load-bearing capacity and shattering into very small fragments and powder, with minimal energy dissipation during the fracture process. In contrast, the optimal single-component NCC-modified group (N2) retained some core fragments, but severe spalling occurred along the edges, accompanied by multiple wide, through-going cracks. This observation suggests that while the nano-densification effect of NCC improved the matrix’s impact resistance, it did not provide the necessary tensile bonding force to bridge cracks, thereby leaving the material’s brittle disintegration nature unchanged under impact waves. Conversely, the failure mode of the optimal single-component PPF-modified group (P3) underwent a significant transformation. The integrity of the specimen was greatly enhanced, exhibiting ductile characteristics—cracked but not disintegrated. The three-dimensional network formed by PPF provided a strong physical bridging effect at the moment of matrix cracking, absorbing a substantial amount of impact kinetic energy and effectively limiting the crushing and disintegration of the concrete. The optimal hybrid group (N2P3) demonstrated the highest level of impact integrity. These specimens not only maintained a nearly intact cylindrical geometric configuration but also exhibited only minimal fine cracks and local micro-spalling. This impressive crack resistance validates the cross-scale synergy between micro-characterization and macroscopic mechanics: the NCC-strengthened interfacial transition zone (ITZ) provided a stable anchoring foundation for the fibers, enabling the PPF to effectively resist pull-out sliding at extremely high strain rates. This transformation shifted the pulverization failure characteristic of the control group into a process of high-energy-dissipating micro-crack evolution.

### 3.3. The Results of NCC/PPF Concrete Microstructure and Material Composition

#### 3.3.1. Microstructure Test Results

**Figure 11:**
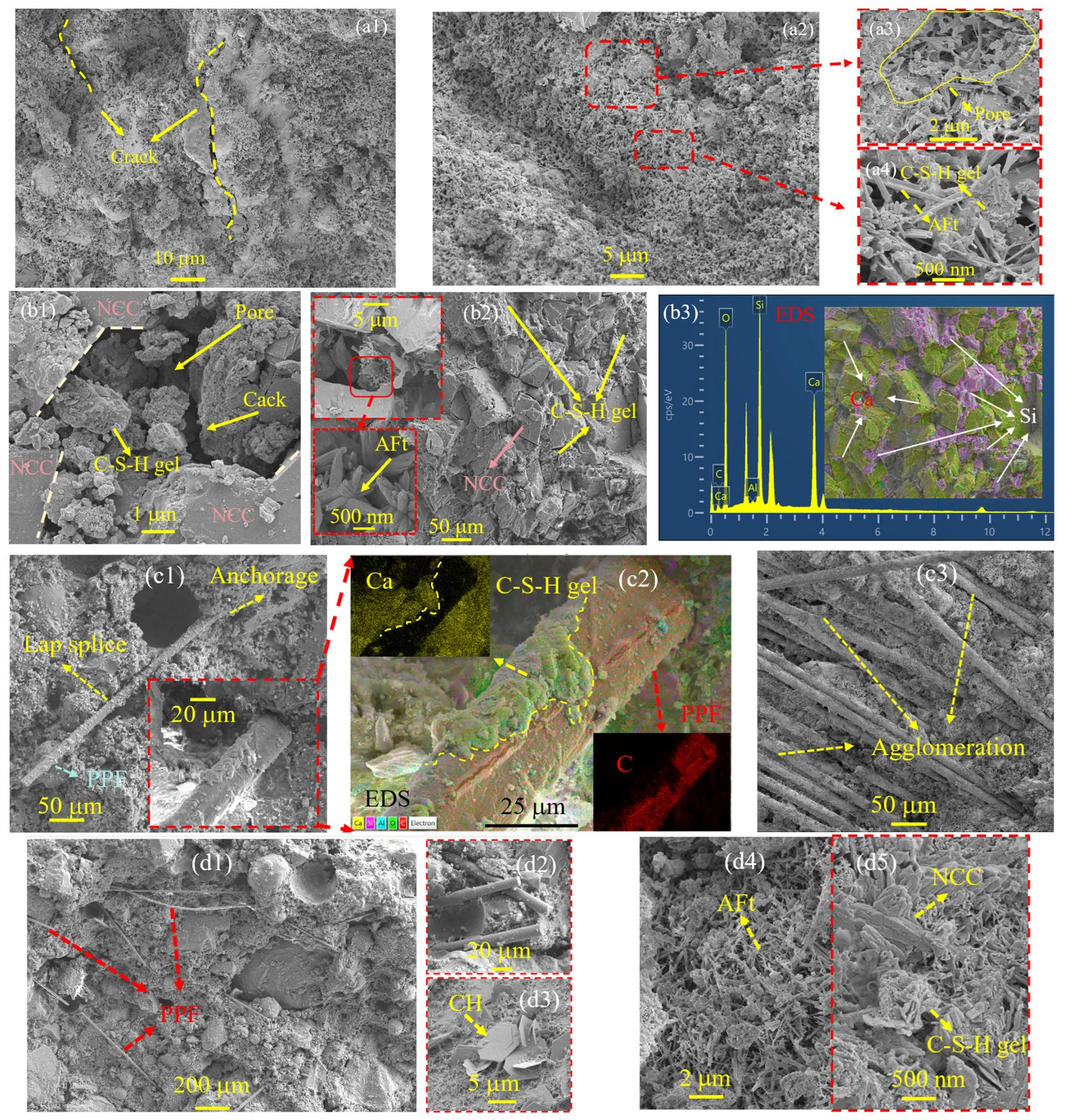
SEM-EDS Test Results of the Sample; (**a1**–**a4**) NP-0; (**b1**–**b3**) NCC concrete; (**c1**–**c3**) PPF concrete; (**d1**–**d5**) NCC/PPF concrete.

Figure 11(a1–a4) illustrates the SEM micro-morphology of the control group NP-0 at various magnifications. Overall, as shown in Figure 11(a1,a2), the internal matrix structure of the specimen is relatively porous, characterised by a rough surface that lacks a continuous dense coating layer, with significant through-going initial micro-cracks evident. Further examination of the localized images (Figure 11(a3,a4)) reveals the presence of large-sized pores within the specimen. Needle-like ettringite (AFt) crystals and amorphous, flocculent calcium silicate hydrate (C-S-H) gels are sporadically distributed around these pores. However, the quantity of these C-S-H gels is relatively limited, and they display a loosely interwoven morphology, which fails to interconnect and form a dense, rigid spatial network. This results in numerous nano- and micro-scale voids between the hydration products. Figure 11(b1–b3) presents the SEM micro-morphology and EDS energy spectrum analysis results for the NCC-incorporated specimen. In Figure 11(b1–b3), fine NCC particles are clearly visible, widely distributed and embedded within the matrix, effectively filling the micro-pores between the cement hydration products. This nano-scale micro-aggregate filling effect significantly refines the internal pore structure, disrupts connected pores, and makes the solid matrix more compact. From the perspective of the nucleation effect, the medium-magnification morphology and its local enlargement (Figure 11(b2)) indicate the formation of numerous dense, blocky, and clustered hydration products within the matrix. The highly active NCC nanoparticles feature a large specific surface area, which reduces the nucleation barrier for hydration products and acts as crystal nuclei for the formation of C-S-H gel. To assess the dispersion morphology of NCC in the final product, EDS elemental mapping was performed. As illustrated in Figure 11(b3), the elemental mapping results indicate that the signals for NCC (Ca regions) and C-S-H gels (Si regions) overlap significantly and are uniformly distributed, with no signs of large-scale agglomeration. This excellent spatial dispersion validates the effectiveness of the ultrasonic pretreatment and reveals the in situ morphology of the NCC within the cementitious matrix, thereby providing a solid structural foundation for the enhancement of macroscopic strength discussed in the following sections.

Figure 11(c1–c3) presents the SEM morphology and EDS elemental distribution results of concrete that incorporates PPF. In Figure 11(c1), individual PPF strands span across micro-cracks or pores within the matrix, with one end deeply embedded in the cementitious matrix. This configuration forms an effective lap splice and anchorage, indicating that PPF primarily exerts a bridging effect within the matrix. The fiber’s ability to achieve optimal bridging efficiency largely depends on the bond strength of the interfacial transition zone (ITZ) between the fiber and the cementitious matrix. The outline of the PPF is clearly identifiable in the carbon (C) element distribution map, while the calcium (Ca) element distribution map, along with the superimposed morphology, reveals a substantial amount of calcium-rich calcium silicate hydrate (C-S-H) gel densely attached to the PPF’s surface (see Figure 11(c2)). This dense coating of hydration products significantly increases the fiber’s surface roughness and frictional resistance, enhancing the mechanical interlock and interfacial bond strength between the PPF and the surrounding matrix. This ensures that the fiber can fully exert its high tensile strength under load. However, when the PPF dosage is excessively high, many flexible fibers experience significant agglomeration (fiber balling) within the matrix. This entanglement of fibers hinders the cement paste from fully penetrating the fiber bundles for encapsulation and hydration, resulting in large structural weak zones and initial bridging voids at the fiber agglomeration sites (see Figure 11(c3)). Figure 11(d1–d5) presents the micro-morphological characteristics of the optimal hybrid group (N2P3). Overall, PPF shows excellent uniform dispersion within the matrix. Many fibers are interwoven, collectively forming a dense three-dimensional spatial crack-resistance network (see Figure 11(d2)). The fiber surfaces and surrounding regions are coated with exceptionally dense hydration products. Additionally, the cementitious system of N2P3 demonstrates a high degree of hydration and spatial densification. In Figure 11(d3), well-developed, large hexagonal plate-like calcium hydroxide (Ca(OH)_2_, CH) crystals are clearly observed in a stacked distribution, indicating sufficient cement hydration. Fine needle-like ettringite (AFt) crystals interweave with substantial amounts of amorphous C-S-H gel, creating a three-dimensional spatial network. The NCC particles, acting as highly active crystal nuclei, are tightly encapsulated at the center by these hydration products, and the nano-pores are nearly completely filled (see Figure 11(d4,d5)).

#### 3.3.2. The Results of XRD Test

**Figure 12:**
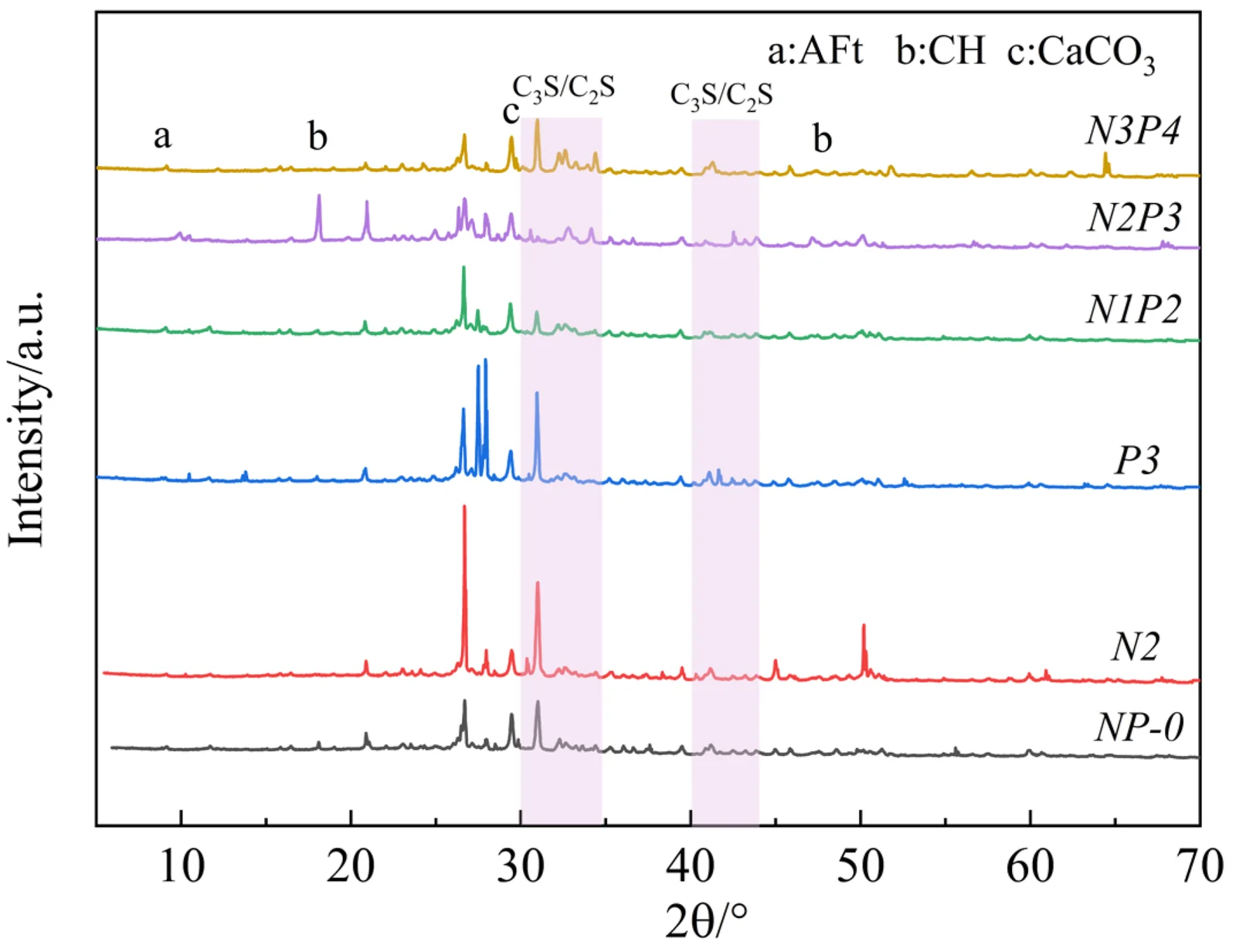
Typical XRD spectrum of NCC/PPF concrete.

Figure 12 presents the X-ray diffraction (XRD) patterns for representative specimens (NP-0, N2, P3, N1P2, N2P3, and N3P4). In general, the positions of the diffraction peaks are consistent across all groups, with characteristic peaks for ettringite (AFt, peak a), calcium hydroxide (CH, peak b), calcium carbonate (CaCO_3_, peak c), and unhydrated clinker (C_3_S/C_2_S) observed in each spectrum. An analysis of the peak intensities among the groups reveals notable evolutionary trends. First, for the groups that include NCC (e.g., N2, N1P2, N2P3, and N3P4), there is a sharp increase in the intensity of the diffraction peak corresponding to calcium carbonate (peak c, 2θ ≈ 29.4°). Second, in the regions of the characteristic peaks for unhydrated cement clinker (C_3_S/C_2_S) (2θ near 32–34° and 41–43°), the diffraction peak intensities for the control group (NP-0) and the single-component PPF-modified group (P3) are relatively high. In contrast, for the NCC-incorporated N2 group and the optimal hybrid group N2P3, the intensities of these unhydrated clinker peaks are significantly reduced. Additionally, the diffraction peak intensities of calcium hydroxide (CH, peak b, near 2θ ≈ 18° and 47°) and ettringite (AFt, peak a) in the N2 and N2P3 groups show varying degrees of enhancement compared to the control group. The significant consumption of C_3_S/C_2_S clinker peaks and the enhancement of CH and AFt hydration product peaks in the NCC-incorporated groups suggest that NCC promotes cement hydration within the system [33]. Additionally, given the inherent reactivity of the P.O 42.5 cement, the inclusion of NCC facilitates a strong chemical activation effect. The high specific surface energy of the nanoparticles considerably reduces the activation energy necessary for clinker hydration, speeding up the interaction between the unhydrated silicate phases and free water. This vigorous chemical reactivity densifies the matrix and optimizes the Interfacial Transition Zone (ITZ) between the cement paste and the PPF additives. As a result, the chemical activation induced by NCC, combined with the physical energy dissipation from the PPF additives, creates a seamless microstructural interaction, resulting in a highly interlocked matrix that enhances both the dynamic and static load-bearing capacities of the composite.

#### 3.3.3. The Results of FTIR Test

**Figure 13:**
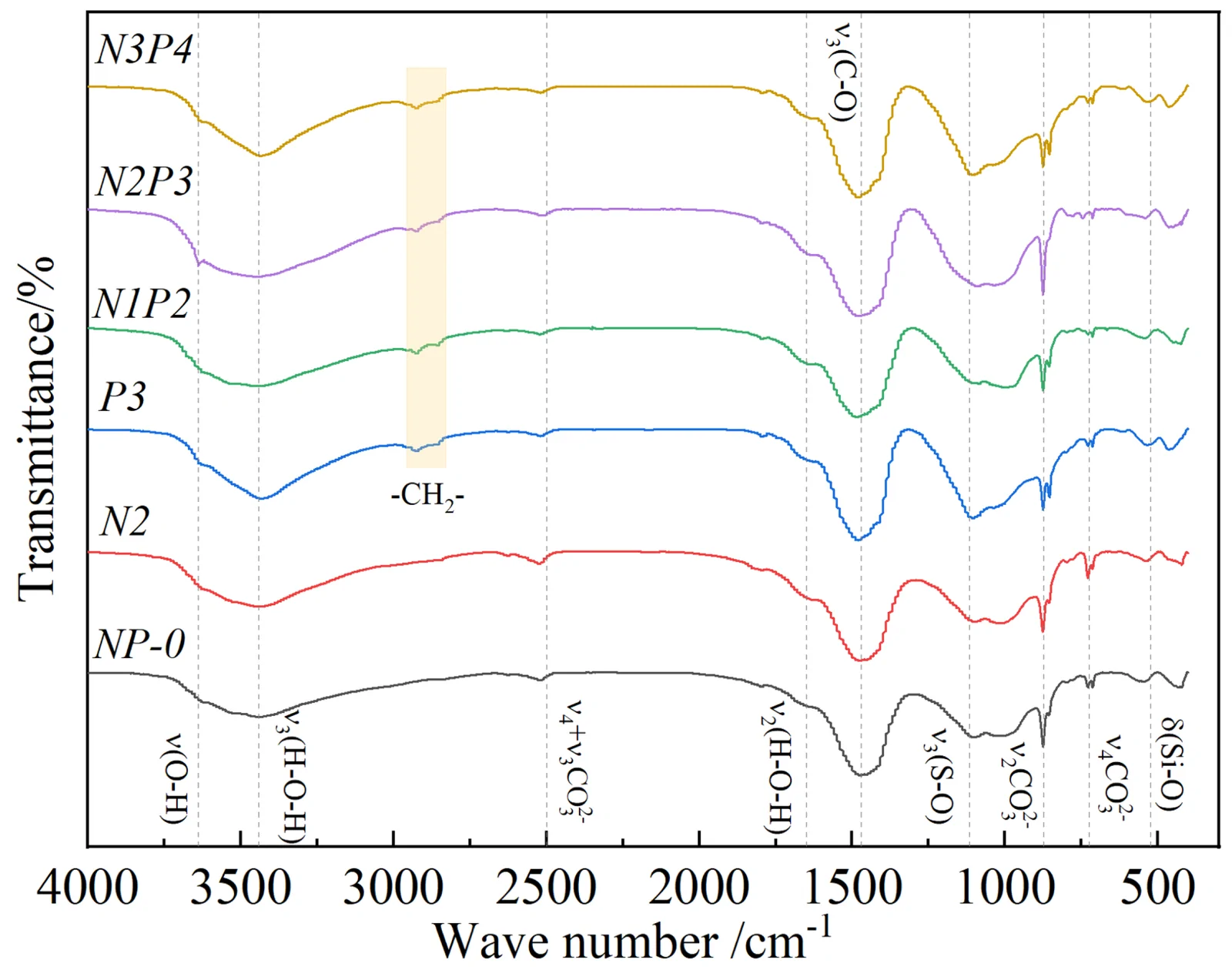
Typical FTIR spectrum of NCC/PPF concrete.

Figure 13 presents the Fourier-transform infrared spectroscopy (FTIR) spectra of typical specimens (NP-0, N2, P3, and N2P3). Overall, the peak positions in the spectra of all groups are generally similar, each containing the *v*(O-H) stretching vibration peak representative of calcium hydroxide (approximately 3640 cm^−1^). The peak at 3440 cm^−1^ is attributed to the *v*3(H-O-H) stretching vibration of bound water in hydration products such as C-(A)-S-H gel and AFt [34], the peak at 1640 cm^−1^ corresponds to the H-O-H bending vibration of bound water, while the absorption peak at 1110 cm^−1^ corresponds to the *v*3(S-O) asymmetric stretching vibration, which may be associated with the presence of gypsum or AFt generated during hydration in the system. The position at 476.3 cm^−1^ corresponds to the Si-O bending vibration absorption peak in [SiO_4_]^4−^ [35], and the typical carbonate *v*3(C-O) and *v*2(CO_3_^2-^) vibration peaks appear at approximately 1450 cm^−1^ and 870 cm^−1^, respectively. A comparison of the spectral details reveals two highly significant phenomena. First, in all groups incorporated with PPF (P3, N1P2, N2P3, and N3P4), distinct -CH_2_- stretching vibration peaks appear at approximately 2926 cm^−1^ and 2850 cm^−1^, which are characteristic of aliphatic compounds [36]. These peaks are completely absent in the control group (NP-0) and the single-component NCC-modified group (N2). Furthermore, compared to the control group, the NCC-incorporated groups (particularly the N2 and N2P3 groups) exhibit varying degrees of enhancement and broadening in the relative intensity and peak area of the absorption peaks representing crystalline and gel water (3440 cm^−1^ and 1640 cm^−1^) as well as the C-O vibration peaks of carbonate.

The evolution of the FTIR characteristic absorption peaks provides verification of the synergistic enhancement mechanism between NCC and PPF at the level of molecular chemical bonds. The presence of aliphatic compounds in the PPF-incorporated specimens, along with the absence of new functional groups in the overall spectra, indicates that PPF relies solely on its geometric morphology and the rough surface gel layer to establish physical anchorage within the concrete, thereby exerting both macro- and micro-scale bridging and crack-resistance effects. In the NCC-incorporated groups, a significant enhancement of absorption peaks for bound water (H-O-H) and free hydroxyl groups (O-H) is observed. This suggests that the introduction of NCC nanoparticles effectively lowers the hydration reaction barrier and promotes the hydration process of silicate clinker, resulting in the formation of C-S-H gels and crystals that contain a substantial amount of chemically bound water within the matrix [37,38]. This NCC-driven chemical densification and PPF-driven physical crack resistance act synergistically, leading to significant improvements in the static and dynamic mechanical properties of NPC.

## 4. Conclusions

This study systematically investigated the cross-scale synergistic enhancement mechanisms of nano-calcium carbonate (NCC) and polypropylene fibers (PPF) on the static and dynamic mechanical properties of concrete. The primary conclusions are as follows:

(1) Optimal Hybrid Proportion and Mechanical Enhancement: The hybrid modification significantly enhances both quasi-static and dynamic properties. The optimal mix proportion was determined to be 1.5% NCC combined with 1.5 kg/m^3^ PPF. This optimal system successfully increases the static compressive strength by 51.56% and the dynamic compressive strength by 62.47%, fundamentally transforming the macroscopic failure mode from brittle pulverization into a high-energy-dissipating ductile deformation.
(2) Micro-Synergistic Mechanism: Multi-scale characterizations confirm a perfect physical-chemical synergy. NCC provides robust chemical activation and nano-filling effects, which intrinsically densify the cementitious matrix and the Interfacial Transition Zone (ITZ). This strengthened ITZ serves as a highly stable anchoring foundation, thereby maximizing the physical crack-bridging and pull-out energy dissipation efficiencies of the PPF network.
(3) Load-Dependent Dominance and Threshold Effect: The synergistic mechanisms exhibit distinct load-path dependency. NCC-driven matrix densification dominates the static bearing capacity, whereas the PPF-driven cross-crack bridging dictates the impact resistance under high-strain-rate dynamics. Furthermore, both modifiers demonstrate a significant threshold degradation effect; exceeding the optimal dosage introduces agglomeration defects, necessitating strict proportion control in practical engineering applications.

## Figures and Tables

**Table 1:** The chemical composition of cement.

Compose	CaO	SO_3_	Fe_2_O_3_	SiO_2_	NaO	Al_2_O_3_	MgO	Other
Content (%)	66.3	2.5	3.5	19.6	0.6	6.5	0.7	0.3

**Table 2:** PPF performance indicators.

Specific Gravity	Diameter/μm	Tensile Strength/MPa	Elastic Modulus	Length/mm
0.91	18~48	>486	>4.8 GPa	6~12

**Table 3:** Physical properties of Nano-CaCO_3_.

Average Particle Size/nm	Apparent Density/(g·L^−1^)	Specific Surface Area/(m^2^·g^−1^)	Content/%	pH
30	300	180	99	9.3

**Table 4:** Concrete mix ratio.

NCC/%	PPF/%	Cement/kg	Fly Ash/kg	Coarse Aggregate/kg	Fine Aggregate/kg	Water Reducer/kg
Table 5	Table 5	461	75	1252	512	2.5

**Table 5:** NCC/PPF Concrete Numbering.

Sample	NCC/%	PPF/kg/m^3^	Sample	NCC/%	PPF/kg/m^3^
NP-0 (175 kg)	0	0	N1P3 (172.13 kg)	1.0	1.5
N1 (173 kg)	1.0	0	N1P4 (171.82 kg)	1.0	2.0
N2 (173 kg)	1.5	0	N2P1 (173.22 kg)	1.5	0.5
N3 (173 kg)	2.0	0	N2P2 (172.52 kg)	1.5	1.0
P1 (173 kg)	0	0.5	N2P3 (172.13 kg)	1.5	1.5
P2 (172.5 kg)	0	1.0	N2P4 (171.82 kg)	1.5	2.0
P3 (172.1 kg)	0	1.5	N3P1 (173.22 kg)	2.0	0.5
P4 (171.8 kg)	0	2.0	N3P2 (172.52 kg)	2.0	1.0
N1P1 (173.22 kg)	1.0	0.5	N3P3 (172.13 kg)	2.0	1.5
N1P2 (172.52 kg)	1.0	1.0	N3P4 (171.82 kg)	2.0	2.0

Note: The mass in parentheses is the mass of water per cubic meter in the mix proportion of this sample.

## Data Availability

The original contributions presented in this study are included in the article. Further inquiries can be directed to the corresponding authors.
